# Nutrition Education in the Australian New South Wales Primary School Curriculum: Knowledge and Attitudes of Students and Parents

**DOI:** 10.3390/children7040024

**Published:** 2020-03-27

**Authors:** Nienke de Vlieger, Jolien van Rossum, Nicholas Riley, Andrew Miller, Clare Collins, Tamara Bucher

**Affiliations:** 1 School of Health Sciences, Faculty of Health and Medicine, The University of Newcastle, Callaghan 2308, NSW, Australia; Nienke.devlieger@newcastle.edu.au (N.d.V.); clare.collins@newcastle.edu.au (C.C.); 2 Priority Research Centre in Physical Activity and Nutrition, The University of Newcastle, Callaghan 2308, NSW, Australia; 3 School of Education, Faculty of Education and Arts, The University of Newcastle, Callaghan 2308, NSW, Australia; 4 Teachers and Teaching Research Centre, School of Education, Faculty of Education and Arts, The University of Newcastle, Callaghan 2308, NSW, Australia; 5 School of Environmental and Life Sciences (SELS), The University of Newcastle, Ourimbah 2258, NSW, Australia

**Keywords:** nutrition, education, knowledge, children, parents, school, dietary intake, attitude

## Abstract

In NSW, Australia, the views of primary-school aged children and their parents in regard to the importance of nutrition education at school are unclear. The aim of the current study was to explore children’s knowledge of nutrition and eating habits and to identify gaps that future school nutrition education programs could target. Students aged 9 to 12 years and their parents (*n* = 21 dyads) were invited to participate in semi-structured interviews, complete a nutrition knowledge questionnaire, and perform a “healthy-unhealthy” food sorting task in a University food laboratory. Among the children, nutrition knowledge scores concerning “serves & portions” of common foods were lowest, identifying a gap in knowledge related to portion size. All children categorized fruits, vegetables, cola, and water correctly as “healthy” or “unhealthy” in the sorting task, but not for the sausage and muesli bar, suggesting that further support categorising processed foods may be needed. The interviews indicated that parents do actively try to teach their children about nutrition, although they reported feeling uncertain about their own level of nutrition knowledge. Children and parents indicated that there is very little nutrition education in school and more is needed. This research could be used to inform future curriculum components related to nutrition education for primary school children.

## 1. Introduction

Good nutrition in children is important for well-being, school performance [1,2], and prevention of chronic diseases such as obesity, type-2 diabetes, and some cancers [3,4]. Additionally, poor dietary behaviours have been shown to track into adulthood, making childhood a critical period in which to establish healthy eating habits [5]. The 2015 NSW School Physical Activity and Nutrition Survey (SPANS) found that only 5% of Australian children consumed the recommended amount of vegetables and approximately 39% of total daily energy was derived from energy-dense, nutrient-poor items (e.g., chips, soda, doughnuts) [6]. This finding is concerning, with the Australian Guide to Healthy Eating (AGHE) recommending that no more than three serves, or roughly 10%–20% (depending on age, height, and activity level) of daily energy consumed, should come from discretionary foods [7]. This dietary imbalance could lead to an excessive energy balance, inadequate nutrient intake [8], and higher intakes of sodium, added sugars, and saturated fat [9].

SPANS 2015 also reported that undesirable eating behaviours such as snacking on unhealthy foods and breakfast skipping are common among children and that these behaviours were more prevalent during adolescence [6]. Whether or not a child adopts healthy eating behaviours and learns about nutrition is dependent on factors such as the household’s socio-economic status [10], parental education level [11,12], parental eating patterns [13], and cultural status [14]. However, when children start attending school, parental influence is replaced by more input from peers and teachers and is more influenced by foods that are available in school [15]. Adolescents generally gain more autonomy over their food choices after the age of 12 [16], with parental control diminishing and peer-pressure in regard to eating behaviours increasing. To prevent poor dietary habits and excessive weight gain, targeting nutrition knowledge among primary school aged children has been proposed as an approach that can contribute to establishing healthy eating habits among adolescents [6,17]. Dudley et al. investigated teaching for the promotion of healthy eating in their meta-analysis review and identified 13 studies with nutrition knowledge outcomes [18]. Eight of the 13 studies reported statistical significances of *p* < 0.001 in improving nutrition knowledge levels, with three displaying large effect sizes [18]. 

While it has been reported that nutrition education interventions can increase children’s nutrition knowledge, the overall evidence base for the impact of nutrition education on dietary behaviours remains weak or unclear [19]. However, in 2002, Worsley [17] examined the available research of the effect of nutrition knowledge on dietary patterns and concluded that nutrition knowledge “may play a small but pivotal role”. In a review by Van Cauwenberghe et al. eight years later, only limited evidence was identified for the effectiveness of nutrition education interventions [19]. Their overall conclusion was that nutrition education, as part of a multi-component approach combined with increased availability of fruits and vegetables and parental involvement, was successful overall and produced the largest effect sizes [19].

Stand-alone, nutrition education has been shown to affect short-term eating behaviour in children. In Ireland, an education program comprising of 20 sessions (10 weeks) displayed significant differences in eating behaviours and preferences after three months in children aged eight to ten years old [20]. Additionally, a study following a cohort of children aged eight to nine years for three years found an overall positive impact on eating behaviours in response to nine to twelve weeks of annual nutrition education, particularly amongst children with lower nutrition knowledge at baseline [21]. 

As schools engage the majority of children, including those who might not have been taught or exposed to appropriate eating behaviours in their home environment [15], they are an ideal setting to effectively teach nutrition education and healthy eating [22,23]. Schools promoting health by adopting a “health promoting school” approach have been found to be of positive influence on their student’s health [24]. Furthermore, a recent recommendation that came forth from the World Health Organization’s report on the Commission on Ending Childhood Obesity stated a healthy school environment must be promoted and advises “integration of nutrition and health literacy and practical skills into the core curriculum” [25]. 

In NSW, Australia, nutrition is described in the syllabus content, however it is included within the broader topic area of “Healthy, Safe and Active Lifestyles”, rather than as an individual topic in the curriculum [26]. Previous research regarding nutrition education in NSW primary schools has demonstrated that teachers who focus on nutrition in class most likely have a personal interest or the school insist on it, with most using self-acquired knowledge rather that any depth of knowledge acquired through teacher education [27]. Investigation of teachers’ time allocation, translation, and attitudes towards nutrition education in primary schools demonstrated that even though teachers might be motivated to teach their students about nutrition, they face barriers such as a crowded curriculum and a lack of appropriate and easily accessible resources [27,28].

As previously stated, nutrition education is most effective when parents are involved [19]. However, it is unclear to what degree parents are involved in or engage with nutrition education programs within primary schools or how the parents feel about nutrition education programs. The current authors previously published an exploratory study about teachers’ attitudes towards nutrition education in schools and the extent to which nutrition concepts are translated into school programs [27]. In order to create a more complete picture of and context for nutrition education in NSW primary schools, it needs to be further examined from the perspective of students and their parents. This will potentially facilitate the development of school-based nutrition education interventions that are acceptable to students and their parents. This is needed to map the current state of nutrition education in NSW primary schools from all perspectives and to establish where opportunities lie for future nutrition education interventions. Therefore, the aim of the current study was to gain insight and find gaps in the average levels of nutrition knowledge of students (year 5 and 6) and their parents. In addition, in an interview with child-parent dyads, attitudes, beliefs, concerns, and desires about nutrition education in schools are explored.

## 2. Materials and Methods

### 2.1. Participants and Procedure

Children in the last stage of primary school (years 5–6; approximately 9–12 years old) were invited to participate in the study and visit the University of Newcastle, Australia during their annual school break, accompanied by one parent. All children attending year 5 or 6 of primary school at the time of recruitment were eligible for inclusion. A convenience sample was recruited via social media and community recruitment flyers, with secondary recruitment via the researcher’s personal network. Parents gave consent for themselves and their child, with children providing assent before commencing involvement in the study. The study started with the parent and child individually filling out a dietary pattern and nutrition knowledge survey. Parents were not allowed to assist their children in answering the nutrition knowledge questions. The survey was administered using Qualtrics survey software (Qualtrics, Provo, UT, USA) on an iPad.

Following the survey, children were asked to sort 17 commonly consumed snacks and beverages into containers labelled “unhealthy”, “neutral”, and “healthy”. Children performed this task without help from their parents or the researcher (dVN), but were asked to think aloud, with their comments recorded by the researcher. 

Finally, a semi-structured interview with both the child and their parent was led by a researcher and lasted approximately 10 to 15 min. The interview was recorded and subsequently transcribed by an independent Australian company (Pacific Transcription, Milton, QLD, Australia). The focus of the interview was the child’s beliefs and attitudes towards healthy eating, the role it plays for the parent at home, and how extensive the school focus is towards nutrition education (child) and nutrition information directed at the home (parent). All procedures were approved by the University of Newcastle Human Research Ethics Committee (Approval number H-2017-0416).

### 2.2. Child Nutrition Knowledge Survey (CNK-AU) Development

The child nutrition knowledge survey that was used in the current study (CNK-AU) was based on an existing Belgian nutrition knowledge survey (CNK-BE), but was adapted for Australian purposes. The CNK-BE was developed by Vereecken et al. [29] and has previously been tested for content validity and reliability using eight experts and 386 children aged 7–12 years. It was reported to be practical, appropriate, and reliable for use in this age group. With permission from Vereecken et al., the CNK-BE was translated into English (dVN) and checked by an independent native speaker (BF). The research team who was involved in adapting the CNK-BE into the CNK-AU were Australian nutrition, dietetics, and education experts, who modified the CNK-BE to align with Australian dietary guidelines, nutrition recommendations, and Australian food culture. Several common Belgian foods and drinks were replaced by similar products that are more commonly consumed by Australian children based on data derived from the Australian Nutrition Survey 2011–2012 [30]. For example, commonly consumed Belgian breakfast foods were replaced with a range of Australian breakfast cereals. Furthermore, the food group categorisation questions were adapted to fit the Australian Guide to Healthy Eating plate model [7]. The reliability of the CNK-AU was tested with 94 children and found to be reliable and a practical nutrition knowledge measurement tool for children aged 8 to 12 years [de Vlieger et al. personal communication 2 July 2019].

The CNK-AU consists of four categories: 1) “healthy food choice” questions (*n* = 11), which present two to four pictures of commonly consumed foods/beverages from which the respondent is asked to identify the healthiest item. Category 2) “serves & portions” (*n* = 11) questions are multiple choice questions about the Australian daily recommended food group serves and balanced meals. The third category 3) “nutritional values” consists of nine multiple choice and two true/false questions regarding functions of nutrients. Lastly, in category 4) “food groups”, participants have to choose which item is the “odd one out” (i.e., belongs to another food group) from four foods or beverages. A total score of 40 points was possible, with responses of “I don’t know” scoring as incorrect and zero points.

The same nutrition knowledge questions were used for both the child and the parent, who each responded individually. While the CNK-BE was not previously tested for reliability or validity with adults, the CNK-AU was completed by parents in the current study to investigate the construct validity of the CNK-AU and to test for correlations with their children’s scores. Several background questions on age, gender, year of school, type of school, technology in the classroom, and lunch at school were included at the start of the survey. 

### 2.3. Dietary Pattern Questions

A short survey (six-questions) on dietary habits was included at the start of the CNK-AU. The questions were taken from a previous study with family dyads [31]. It included five multiple choice questions and one matrix question regarding the frequency of their consumption of fruits, vegetables, take-away meals, chips, candy, biscuits, drinks, and foods from the remaining food groups (i.e., grains, meats, etc). The parents and children self-reported on these questions individually. The complete list of dietary pattern questions is summarised in supplementary Table S1.

### 2.4. Sorting Task

Children participating in the study were asked to sort 17 foods/drinks into boxes marked “Unhealthy”, “Neutral”, and “Healthy” (see Table 1). The foods that were used were selected by a team of nutrition experts and were based on data on commonly consumed foods and drinks by Australian children [30]. For most of the foods and beverages, realistic looking validated food replicas were used [32]. The children received no help or feedback from the researcher or parent for the sorting task, but were told what the food/beverage was if they asked or were uncertain. Children were asked to think aloud and to try to give explanations on what they were thinking while sorting. Comments and final sorting results were recorded by the researcher. The sorting task score was calculated by summing the total number of correct answers, with the spoken comments summarised into keywords.

The categorisation of the foods and drinks was based on the recommendations and guidelines of the Australian Dietary Guidelines for children [33]. Table 1 shows the categorisation.

### 2.5. Interviews

In order to collect exploratory data on the children’s and parent’s personal experiences, attitudes, perceptions, and beliefs concerning nutrition education, the last part of the study involved semi-structured interviews. Supplementary Table S2 lists all the interview questions. The interviews were audio recorded and lasted up to 15 min. Several questions were developed specifically for the children (e.g., “Do you think learning about food and what might be good for you or not good for you is important? Why (not)?”). Some questions were only asked of the parents (e.g., “Do you think teaching your child (ren) about nutrition is important?”) and some questions could be answered by both (e.g., “Do you have any other ideas or suggestions for activities/tasks/materials in terms of nutrition education?”). Follow-up questions to clarify responses were asked where necessary. A checklist was used to ensure all questions had been asked in a standardised way by the end of the interview. Interviewees were allowed to share their thoughts about other issues if time permitted. This allowed the researchers to collect information beyond the set questions and themes on issues of importance to the parents and children.

### 2.6. Statistical Analysis

The software IBM SPSS Statistics (IBM Corp., version 25, Armonk, NY, USA) was used to analyse the data. Means (*M*) and standard deviations (*SD*) were used to report quantitative and descriptive statistics. Dependent sample t-tests were used to compare children’s nutrition knowledge scores with those of their parents. Counts of correct answers and keywords for the sorting task were analysed using Microsoft Excel (Microsoft Excel for Office 365 MSO, Version 1911).

The digital audio files from each interview were transcribed verbatim. The lead author read the transcript and listened to the audio recording to become familiar with the data. Each transcript was then divided and copied into separate excel sheets, representing the children’s conversations and those of the parents. Both files were coded thematically, using an open coding process, whereby meaningful quotes or key examples from participants were extracted and sorted together. These grouped quotes and key examples were used to generate themes and draw conclusions as to the dominant attitudes and opinions. A second co-author (NR) provided critical feedback on the analysis and interpretations of the interview data.

## 3. Results

### 3.1. Descriptive Statistics

Descriptive statistics are summarised in Table 2. In total, 21 child-parent dyads participated. The average child age was 10.3 (*SD* 0.6) years and 43.1 (*SD* 4.6) years for parents. Five children and two parents were male. The majority (86%) of children attended year 5 and most were enrolled in a government public school (81%). Using residential postcodes reported by the parents, an average score of 7.7 was found for socio-economic status on a scale of 1 to 10, with a higher value indicating a relative socio-economic advantage [34]. All children had at least one other sibling living at home and only one parent had part-time care for their child. 

### 3.2. CNK-AU Scores

Out of a total possible 41 points for nutrition knowledge, children scored a mean of 22.3 (*SD* = 0.92) and parents 31.0 (*SD* = 2.85) (see Table 3). The difference between child and their parents’ total score was significant: *t*_(20)_ = −7.63, *p* < 0.05. Only the category of “healthy choices” in the nutrition knowledge survey was answered similarly by both the parent and their child (*t*_(20)_ = −1.82, *p* > 0.05).

### 3.3. Dietary Patterns

In the sample of dyads in the current study, it was found that parents report eating more serves of vegetables (*M* = 1.76, *SD =* 1.77) compared to their child’s self-reported intake (*M* = 0.94, *SE* = 1.6). The difference was statistically significant *(t*_(20)_ = 2.4, *p* < 0.05), with the difference in fruit consumption not significant (*M_parent_* = 1.81, *M_child_* = 1.67, *t_(20)_* = −0.72, *p* > 0.05). 

Other significant differences in consumption frequencies for parent-child were for candy (*t*_(20)_ = −2.28, *p* < 0.05) and soda (*t*_(20)_ = −2.03, *p* < 0.05). In both cases, parents reported consuming these items more often than children. For consumption of water, milk, meats and fish, bread, potatoes and pasta, biscuits, and diet soda, no differences in consumption were identified between children and parents. 

Most of the parents reported that they eat: “less than one serve of fruit per day” (43%); “One serve of vegetables per day” (48%); have take-away meals three times per month or less (86%); and eat candy less than once per month or never (81%). Most children reported that they eat: less than one serve of fruit per day (29%) or never eat fruit (38%); one serve of vegetables or less per day (76%); have take-away meals three times per month or less (76%); and have candy one to three times per month (48%). Most children believed that their diet was healthy (67%) and of the parents, 71% believed that the food they eat was healthy. Among the parents, 100% said they like eating healthily, while only 86% of the children reported liking it. The complete results of the dietary pattern questions are summarised in supplementary Table S1.

### 3.4. Sorting Task Children

After completing the nutrition knowledge survey, the children completed a sorting task. Results of the sorting per food/beverage are summarised in Table 4. 

Out of the 17 foods, on average, 11.6 (SD = 1.86) snacks were sorted correctly per child. All children put the water, banana, carrot, and cola in the correct box. However, both for the sausage and muesli bar, only three children put the food item correctly in the unhealthy box. Comments made by the children about why they sorted a food or beverage into “healthy”, “neutral”, or “unhealthy” are summarised in Table 5. “Fruit” and “vegetables” were mentioned most frequently as criteria to indicate healthiness (19 times), while “sugar” was used 51 times to describe why a food/beverage was unhealthy. Identifying why foods/beverages were identified as being “neutral” was more difficult for the children and was the most frequently reported assessment.

Thirteen of 21 participants checked a food label at least once (seven included products had a label). The most frequently mentioned reasons for marking food items as unhealthy were based on sugar content (mentioned 51 times) and the food item containing chocolate (mentioned 14 times). The most frequently mentioned reasons for sorting healthy foods were based on the food group it belonged to; fruit and vegetables (mentioned 19 times). 

### 3.5. Interviews

A full list of the interview questions are located in supplementary document S2.

#### 3.5.1. Children’s Beliefs about Healthy Eating and Nutrition Knowledge

All children stated that it is important to eat healthily, with explanations varying from statements about how healthy food nourishes your body, e.g., “*it is good for your body*” and “*you need to eat a good balance*,” to statements focusing more on negative aspects, e.g., “*if you don’t eat healthy, you can turn out basically the size of half this room,” and “if you eat unhealthy you can get diabetes”*. Eating in a balanced manner and not having to always eat healthily were reported several times.

Seventeen children mentioned at least once that it is important to eat healthily or to know about eating healthy in order to avoid becoming overweight or developing a disease and/or dying. Several of the children also referred to their future as a reason to learn about nutrition:

“Yes, so then we can know what’s healthy and what’s unhealthy. For when you grow up.”

—Girl, 10 years old

Another reason that was often given for learning about nutrition was so you can “be healthy” and “grow well”:

“Yes, it is good to learn about nutrition. Because at this age you’re at a growing point and if you don’t eat healthy, then you won’t be able to grow that well.”

—Girl, 10 years old

#### 3.5.2. Nutrition Education and Health Behaviours at Home

Twenty dyads confirmed that nutrition or healthy/unhealthy foods were discussed at home at least sometimes. This ranged from explaining and/or teaching their children to simply ensuring that the children have more access to healthy choices, e.g., by cooking healthy food or providing healthy school snacks. Several dyads mentioned a more “teach as you go” approach:

“Sometimes if I go to eat something it’ll be like do you need that sort of thing. I could make a better choice.”

—Boy, 11 years old

While not all children indicated that they enjoy helping their parent(s) shop and cook, they all said they do both; either sometimes or frequently.

All parents said they believe it is very important to address nutrition at home and most parents mentioned healthy eating as a central standard observed in their household. Most parents mentioned that they believe it is important to ensure their child will have the skills and knowledge to make the right choices, as opposed to simply providing the healthy option for them. For example, by teaching them how to make sense out of conflicting information that their child might be exposed to and how to make the right choice:

“Absolutely. Because there’s so much—so many—they get so much different information that’s conflicting […]”

—Mother of 11 year-old boy

“Absolutely. I think it’s important to start early so that they make the right choices when they’re older themselves. I can help them make choices now but when they’re older they need to know.”

—Mother of 10 year-old girl

“[…] so sort of just teaching a little bit more about investigating. Making up your choice, not just believing what is on the front but digging a bit deeper.”

—Mother of 10 year-old boy

“Definitely so they can make the right choices and also understand why as a parent you’re setting particular boundaries and instead of giving them certain things, if they understand why you’re doing it and what purpose they’re more likely to get on board rather than just say why do you keep denying me all these things?”

—Mother of 10 year-old girl

Furthermore, most parents pointed out how that they try to talk about the foods *they* choose to eat at home or when a food is an “occasional” choice and what makes a food unhealthy:

“But often we just talk about—you know, like we might be out and some children might be eating some certain foods and my children might want to have it and when things like that come up we would have a bit of a talk about it and why do we believe that we don’t want our children to have it.”

—Mother of 10 year-old girl

All parents said that they felt confident to teach their child about nutrition, but 18 of them also added that they do not know everything and felt there “might be a few gaps”. Nine of the parents mentioned that they were interested in nutrition themselves.

#### 3.5.3. Parental Involvement and Nutrition Education in the Child’s School

Overall, 15 of 21 children said that they did not recall ever having been taught about nutrition, except for recalling having seen Healthy Harold (a mobile health-educator, popular in Australian schools) or some other basic nutrition concepts in kindergarten or year 1. The rest of the children recalled some information about nutrition, but it was either negligible (e.g., “one hour per term” or “one persuasive writing activity”) or focused on only one aspect of nutrition (e.g., food packaging, allergies). 

One child participant mentioned her teacher “*is healthy and she encourages some kids”* and that there is a lot of discussions about food in class. Two children mention another teacher coming in specifically for teaching nutrition, but one of them mentioned that the lesson was boring:

“Whenever the teacher comes around it’s pretty much the exact same thing every time. They go over everything and then just keep reminding you every so often. You’re just hearing the same thing over and over. Boring yeah.”

—Boy, 11

Only four of 22 parents indicated that they received some information about nutrition (e.g., healthy lunch boxes) from the school, but only one participant was positive about this. Two mentioned that it was only once-in-a-while and one found it was “not useful at all”. The rest of the parents said they had not received any information to date, but eight had received something or attended a session at school when the child was in kindergarten. Several dyads noted that there was some information on fruit breaks (Crunch ‘n Sip), but that it was not necessarily informative. Seventeen of the parents would be happy to receive more information about lunchbox ideas or other nutrition information in the future.

*“No the only thing I can think of is when they’re starting kindergarten, they do talk about making good choices in lunchboxes and five serves of veggies and two fruit, but then that’s kind of—that’s sort of given to you—well it’s really a couple of months before they start kindergarten. Then there’s not really any follow up on that and. Yeah, lunchboxes are hard because of just time stuff. You’re wanting to rush and it’s easy to make three sandwiches in the morning than make salads.”*

—Mother of 10 year-old girl

Nineteen dyads responded affirmatively when asked if they would like to see more nutrition education in school. Some examples:

“I even just think more just the science behind healthy eating, so I’d love them to actually in school talk about what is calcium, what are vitamins, what is the diseases in Australia, what food helps eyesight, what food helps bones, what food. So I don’t see a general primary school teacher having time in the curriculum to do that, but I think it should. The only time there’s a little bit of influence in the school is when it comes to her lunchbox, which puts it back on the parent.”

—Mother of 10 year-old girl

“Yeah, I think it would be good for everyone knowing it—as well as it being funny we’re also learning about it and learning what’s in the food that they’re making. Maybe a bit more about the diet and what sort of—mum and dad have mentioned before the type of fruit or vegetables, how much you need to eat but maybe it would be better to be reminded.”

—Girl, 10

“If they actually taught us different stuff. If it wasn’t boring.”

—Boy, 11

One child said no because *“I’ve been lectured about it at home too much!”* but did recognise that it might be beneficial for classmates. Another parent thought that *“It’s not the teacher, I think* [the children] *just need to listen a bit more”*.

Others have also expressed their dissatisfaction with the teacher or school using unhealthy foods as rewards and one participant thought that more nutrition education could help with that as well:

“Well yeah, a little bit more because sometimes we have—if we do something good or something in class we get these little Starburst candy they give out.”

—Girl, 10

In addition, the children were asked how they would like to see nutrition education presented in their classroom. Only a few children had any ideas and mentioned more interactive learning, keeping food diaries, group projects, and working with props. 

## 4. Discussion

The main objective of the current study was to map the current nutrition knowledge of children and their parents, identify any gaps in their knowledge that need attention, and to investigate their attitudes towards nutrition education. This was done via a sequence of steps; a dietary intake and nutrition knowledge survey, a sorting task by the children, and a semi-structured interview. 

On average, children correctly answered half of the questions in the CNK-AU. This is very similar to the reported 51% found for the CNK-BE [29]. As expected, parents scored significantly better than children, answering about three quarters of the questions correctly. The nutrition knowledge survey highlighted gaps in the children’s knowledge that could be addressed in future nutrition education programs. In particular, the nutrition knowledge categories concerning daily recommended food group serves and the specific functions of nutrients scored low. Similar poor results were identified for knowledge of food functions in an American nutrition knowledge survey among children six to 10 years, with the researchers reporting the lowest test scores for their questions on food functions [35]. In the current study, all children knew that milk was important to build strong bones, while less than one third knew what the functions of vitamins, fibre, carbohydrates, or meat were. 

Furthermore, the child participants who completed the nutrition knowledge survey were unable to correctly answer questions about the number of daily recommended food group serves they need and found it difficult to describe what constitutes one serve of each food group. The Australian Dietary Guidelines are designed to promote health and well-being across the population and to reduce the risk of diet-related conditions and chronic diseases [36]. Adopting a dietary pattern aligned with these guidelines can promote healthy eating and the prevention of obesity among Australians [36]. However, if children do not receive nutrition education in regard to these national guidelines, they will remain unaware of recommendations to follow them. Unfortunately, the questions concerning dietary patterns in the current study indicate that results reflect what is already known about the current dietary patterns of Australian adults and children [6]. On average, both the children and parents reported not consuming adequate daily serves of vegetables and fruit. The mean of only 0.63 serves of fruit and 0.50 serves of vegetables per day for children is substantially lower than the recommended two and five serves of fruit and vegetables per day [7], and results are lower than intakes reported among primary school aged children in the 2015 SPANS [6]. Numerous studies have identified that improving nutrition knowledge can contribute to improving food behaviours, even if the effect size is small [17,18,19]. Therefore, addressing these gaps in children’s knowledge may represent a small but important contribution to improving overall dietary patterns [17]. 

The sorting task, performed by the children only, identified some further knowledge gaps concerning ability to identify “healthy” and “unhealthy” foods and beverages. Some foods that are very familiar to children were sorted correctly by all children (e.g., coca cola, water, banana). However, other products had a higher percentage of children sorting them incorrectly, which could be explained by conflicting information in the environment regarding the product. For example, the processed sausage and muesli bar were correctly sorted least often. While the Australian Dietary Guidelines classifies processed meat as a discretionary food and thus it belongs in the “unhealthy” container [7], some children mentioned “meat is healthy” during the think out loud procedure. Not all foods can be categorised distinctly as either totally healthy versus unhealthy and this concept is something that children find difficult and hence, would potentially benefit from learning more about. Whether a product is considered “healthy” or “unhealthy” also depends on the ingredients and will vary among similar products in the category. For example, not all muesli bars have the same nutrient profile. Reading the nutrition information panel on the food label is one method of helping to decide an item’s healthfulness and hence, which container it should be sorted into. Thirteen of the 21 child participants consulted the nutritional information panel on the product while sorting it. Of those 13 participants, only eight sorted the food or beverage correctly after checking the nutritional information panel. These outcomes highlight the gap in children’s knowledge and ability to read and interpret nutrition information. Food label literacy is an important part of applied nutrition knowledge and it has been shown that being aware of a product’s nutrient composition can affect children’s dietary intake [37].

With regard to the accuracy of knowledge within the sorting task, the current study raises some concerns, echoing findings from Hart et al., where children were asked to sort food as “good” or “bad” [38]. In the current study, many of the children put the white bread slice in the same category as the wholegrain one, indicating a lack of knowledge about the benefits of wholegrain products. Many of the children also seemed to utilise their prepossessing knowledge to sort the foods that might not be accurate or applicable. For example, “contains watermelon” was used to decide that a food was healthier than it actually was. De Vlieger et al. also found that a small percentage of fruit added as an ingredient into an otherwise unhealthy snack led to young adult participants rating snacks as being more nutritious than they actually were [39]. 

The interviews framed the attitudes and beliefs about nutrition and nutrition education. It was clear that most participants value healthy eating and while some children indicated that they also value it, many also noted that they eat healthy because that is “simply the way they eat at home”. Many of the children mentioned becoming “overweight” or diet-disease links when asked why they think they should eat healthy. Hart et al. found that their participants could correctly identify “energy-dense” and “fattening” foods [38], however eating a balanced diet was also frequently mentioned in the current study. This finding is important as it shows that nutrition knowledge may need to encompass more than simply being able to state the function of a nutrient. Topics such as balanced diets, diet-disease relationships, and potentially body image education should be included to respond to children’s easily influential notions on nutrition and healthy eating. 

When asked about their teacher discussing nutrition in class, most dyads mentioned that there was some basic information in kindergarten for both the child and the parent, but in the later primary years, almost nothing concerning nutrition was mentioned. This confirms the findings of a previous study by the current authors, where teachers indicated not spending much time on nutrition in class [27]. All children indicated, however, that they would like more nutrition education and named several teaching methods such as interactive activities, keeping food diaries, and group work as the most fun way to learn about nutrition. Previous research identified integration with other core subject areas (e.g., mathematics) and gamification using smart technologies as methods to provide real life context and make nutrition education more fun [40].

The parents in the current study seemed to make the effort to actively teach their child about nutrition and about healthy or unhealthy choices. All children indicated that they help with cooking and shopping, which parents reported they used as a teaching opportunity. In the literature, multiple studies have found a significant relationship between a mother’s nutrition knowledge and their child’s dietary patterns [41,42,43]. In general, parents are the gatekeepers of their children’s health and, therefore, play an imperative role as models for their children [44]. However, almost all parents say that while they feel confident in their ability to teach their children about nutrition, they believe they do not know everything or that they have the correct knowledge. Not all children will therefore learn accurate nutrition information from their parents, nor will all children grow up with optimal parental role-models for healthy eating behaviour [45]. In addition, while not addressed in the current study, apart from education from their parents, children’s eating behaviours are also influenced by their peers, community, advertising, and the media [15,46]. 

These results highlight the need for evidence-based nutrition education programs and curriculum content in the primary school curriculum. However, De Vlieger et al. found previously that primary school teachers reported that they lacked the time, resources, and knowledge they needed to teach their students about nutrition [27]. A similar study in Victoria, Australia found that in order to teach nutrition education, teachers indicated that they needed ready-to-go resources that are engaging, age appropriate, interactive, authentic, editable, adaptable, aligned with the curriculum, credible, and free or low cost [28]. In the current study, child participants also indicated that they would like nutrition education materials presented in an engaging method. Previous research identified that interactive and cross-curricular methods were best for teaching children about healthy eating [18]. Using these methods, there are numerous possibilities to address the current nutrition knowledge gaps within the curriculum. To ensure high quality and centralised nutrition education for all children, it is proposed that an interactive, standardised curriculum-based nutrition education program be developed by nutrition and education experts. This could be used in schools to ensure all children receive not only nutrition education, but also the most accurate information. 

Some limitations to this study should be mentioned. As this was an explorative study, only a small sample was used. Furthermore, the nature of the research topic and recruiting methods might have led to a bias in recruiting more people with a personal interest in health and nutrition. Based on residential postcodes, the participants recruited were found to predominantly reside in areas of a higher socio economic advantage, which is associated with healthier eating patterns [45], and to have higher levels of nutrition knowledge [10]. The sample is therefore not representative of the whole population and in other population groups with specific needs, additional gaps and issues are likely to be identified. Lastly, results based on the interviews conducted with the children and parents should be interpreted with caution. While it was concluded that children do not receive any, or very little, nutrition education in schools, this is based on children’s recall and their recognition of what they consider nutrition education. Hence, this may not accurately reflect what was taught.

The current study is the first to explore the nutrition knowledge and attitudes and beliefs of Australian children and parents using an exploratory design and short interviews. It was found that there is room for improvement in children’s nutrition knowledge, with programs and curriculum content designed to address the gaps in knowledge that were identified. These gaps included lack of knowledge about recommended daily food group serves, food functions, and interpreting nutrition information panels on food labels. While new nutrition education programs or resources have the potential to be integrated into school curricula and could prove beneficial to children’s knowledge and eating patterns, the current study highlighted a lack of nutrition information in classes or being provided to parents currently. The results from the current study could inform future research in schools, particularly in developing and testing the impact of curriculum-based nutrition education programs on child and family nutrition knowledge, dietary intake, and nutrition related health and well-being. Testing such a program could inform future curriculum design and potentially health policies related to schools.

## Figures and Tables

**Table 1:** Sorting foods in the right box, correct answers based on National Health and Medical Research Council (2013).

Food Item	Correct Box	Based on
Water *	healthy	The Australian Dietary Guideline advice to drink plenty of water.
Plain yoghurt	healthy	Guideline 2: Enjoy a wide variety of nutritious foods from the food group consisting of milk, yoghurt, cheese, and/or their alternatives, mostly reduced fat.
Mixed nuts *	healthy	Guideline 2: Enjoy a wide variety of lean meats and poultry, fish, eggs, tofu, nuts and seeds, and legumes/beans.
Wholemeal bread slice *	healthy	Guideline 2: Enjoy a wide variety of nutritious foods from grain (cereal) foods, mostly wholegrain and/or high cereal fibre varieties, such as breads, cereals, rice, pasta, noodles, polenta, couscous, oats, quinoa, and barley.
Banana *	healthy	Guideline 2: Enjoy a wide variety of nutritious foods from the fruit food group.
Carrot	healthy	Guideline 2: Consume plenty of vegetables, including different types and colours, and legumes/beans.
Egg *	healthy	Guideline 2: Enjoy a wide variety of lean meats and poultry, fish, eggs, tofu, nuts and seeds, and legumes/beans.
Strawberry milk	neutral	Guideline 3a: limit foods high in saturated fat and added sugars. Guideline 2: Enjoy a wide variety of nutritious foods from the food group consisting of milk, yoghurt, cheese, and/or their alternatives, mostly reduced fat.
Orange juice	neutral	According to guideline two of the Australian Dietary Guidelines, people should enjoy a wide variety of fruits. ½ cup of fruit juice (with no added sugar) can only occasionally be considered a standard serve of fruit. Therefore, orange juice is placed in the neutral group.
Straw. yoghurt	neutral	Guideline 3a: limit foods high in saturated fat and added sugars. Guideline 2: Enjoy a wide variety of nutritious foods from the food group consisting of milk, yoghurt, cheese, and/or their alternatives, mostly reduced fat.
White bread slice *	neutral	Guideline 2: Enjoy a wide variety of nutritious foods from grain (cereal) foods, mostly wholegrain, and/or high cereal fibre varieties.
Cola	unhealthy	Considered unhealthy as the Australian Dietary Guidelines state to limit intake of foods and drinks containing added sugars such as sugar-sweetened soft drinks.
Chips *	unhealthy	Guideline 3a: Limit intake of foods high in saturated fat such as many biscuits, cakes, pastries, pies, processed meats, commercial burgers, pizza, fried foods, potato chips, crisps, and other savoury snacks.
Muffin *	unhealthy	Guideline 3a: limit foods high in saturated fat and added sugars.
Timtam *^†^	unhealthy	Guideline 3a: limit foods high in saturated fat and added sugars.
Pork sausage *	unhealthy	Guideline 3a: limit intake of foods high in saturated fats such as meats.
Muesli bar	unhealthy	Guideline 3a: limit foods high in saturated fat and added sugars.

* Replica food; ^†^ An Australian chocolate coated cookie.

**Table 2:** Descriptive statistics.

	Children	Parents
Number of participants	21	21
Mean age (years)	10.3 (*SD* 0.6)	43.1 (*SD* 4.6)
Gender		
Male	5 (23.8%)	2 (9.5%)
Female	16 (76.2%)	19 (90.5%)
Year of school		
Year 5	18 (85.7%)	N/A
Year 6	3 (14.3%)	
Number of other children in the home		2.4 (*SD* 0.8)
Type of school	Government: 17 (81%)	
	Catholic: 3 (14.3%)	
	Independent: 1 (4.8%)	
Average SEIFA * per postcode	7.7	

* Socio-Economic Indexes for Areas (SEIFA) [34]. A value closer to 1 indicates a relative socio-economic disadvantage; a value closer to 10 indicates a relative socioeconomic advantage. Calculated by reported residential postcode.

**Table 3:** Nutrition knowledge survey scores.

	Score Possible	Score Mean ± *SD*		Δ Score Children-Parents	Correlation (*p* Value)
		Children	Parents		
Healthy choices	12	8.24 (0.86)	9.20 (1.44)	−0.95	0.084
Serves & portions	11	4.05 (1.77)	5.67 (1.49)	−1.62	0.001 *
Nutritional values	11	5.67 (2.03)	9.86 (0.96)	−4.19	<0.001 *
Food groups	6	4.33 (0.86)	5.33 (0.80)	−1.00	0.001 *
Total score	40	22.3 (4.22)	31.00 (2.85)	−8.71	<0.001 *

* The difference between the child’s and their parents’ score is significant.

**Table 4:** Results of sorting food items and beverages (*n* = 21).

Food/Beverage	Unhealthy	Neutral	Healthy	% Correct
Banana	0 (0%)	0 (0%)	21 (100%) *	100
Carrot	0 (0%)	0 (0%)	21 (100%) *	100
Chips	17 (81%) *	4 (19%)	0 (0%)	81
Cola	21 (100%) *	0 (0%)	0 (0%)	100
Egg	0 (0%)	4 (19%)	17 (81%) *	81
Mixed nuts	0 (0%)	8 (38%)	13 (62%) *	62
Muesli bar	3 (14%) *	16 (76%)	2 (10%)	14
Muffin	19 (90%) *	2 (10%)	0 (0%)	90
Orange juice	9 (43%)	11 (52%) *	1 (5%)	52
Plain yoghurt	0 (0%)	15 (71%)	6 (29%) *	29
Pork sausage	3 (14%) *	17 (81%)	1 (5%)	14
Straw. yoghurt	5 (24%)	14 (67%) *	2 (10%)	67
Strawberry milk	15 (71%)	6 (29%) *	0 (0%)	29
Timtam	20 (95%) *	1 (5%)	0 (0%)	95
Water	0 (0%)	0 (0%)	21 (100%) *	100
White bread slice	2 (10%)	16 (76%) *	3 (14%)	76
Wholemeal bread slice	0 (0%)	6 (29%)	15 (71%) *	71

* Correct answer.

**Table 5:** Frequency of reasons given for sorting a food/beverage by the child participants (*n* = 21).

Healthy Criteria Mentioned		Neutral Criteria Mentioned		Unhealthy Criteria Mentioned	
Fruit and/or vegetables	19	Neutral	16	Sugar	51
Healthy	10	Sometimes	5	Contains chocolate	14
Natural (sugar)	7	Not healthy/unhealthy	4	Unhealthy	13
Grains	5			Salt	4
Protein	5			Processed	3
Good for you	4				
Vitamins	2				

## Supplementary Materials

The following are available online at https://www.mdpi.com/2227-9067/7/4/24/s1, Table S1: Dietary intake frequency questions; Table S2: Semi-structured interview questions. Differentiated by child and parent.
