# Supplementary material for: Nutrition Education in the Australian New South Wales Primary School Curriculum: Knowledge and Attitudes of Students and Parents
Source: Children (Basel). 2020 Mar 27;7(4):24. doi: 10.3390/children7040024 (PMC7231028; doi:10.3390/children7040024)
Supplement: Supplementary file 1 [file children-07-00024-s001.zip › Supplementary document S2.docx]

**Supplementary table S2:** Semi-structured interview questions. Differentiated by child and parent.

Questions for: child, parent+child, parent

**Aim: Icebreaker/review of tasks and survey done**

- What did you think of the fake food we use here?
- What did you think of the tasks? Did you like them? Did you think they were hard or easy?

(backup question: ‘what is your favourite/least favourite food?)

**Aim: Find out what they learn, how they learn it, what they think of it**

**Aim: How much are the parents involved in food choice, attitudes, nutrition education**

- Here we think learning about healthy or unhealthy foods is very important. Do you think learning about food and what might be good for you or not good for you is important? Why (not)?
- Do you ever talk about it at home? When and how do you talk about it? If no, why not?
- Do you maybe ever help cook at home? Or go to the shops and see what foods you can get there?
- Do you think teaching your child(ren) about nutrition is important?
- Do you get any information regarding nutrition from school?
- What kind of information (how) would you like to get?

**Main aim: Find out where the child gets her/his nutrition knowledge and their general attitude toward nutrition education.**

- Do you ever talk about or discuss food at school? Do you for example talk about what is a healthy or unhealthy food?
- What do you talk about in school? (For example: fast foods, variety of food choices, food choices for good health, effects of food on the body, energy intake/expenditure, salt, sugar, fats, portion size, knowing what a healthy and unhealthy food/drink is)
- Does your own teacher teach you about nutrition? Or does someone else do it?
- Are there any activities/tasks/materials that your teachers discusses or gives to you? (For example, food diary, school garden, video/film, book/magazines, websites, make real foods, excursions, presentation by the students)
- What activities/tasks/materials in the field of nutrition education do you like the most?
- Do you have any other ideas or suggestions for activities/tasks/materials in terms of nutrition education?
- Do you know this? [show AGHE] If yes, can you explain to me how to use it?
- How much time does the teacher usually spend on teaching about food?
- Do you like what you talk about in school? And do you like how you learn about it?
- What else do you think would be useful to learn about food/nutrition? What more could you do at home or school?
- Would you enjoy it if it was in the form of a game/video or uses realistic foods that you can play with and it being more hands-on learning? Maybe something like we did today?
