# Supplementary material for: Nutrition Education in the Australian New South Wales Primary School Curriculum: Knowledge and Attitudes of Students and Parents
Source: Children (Basel). 2020 Mar 27;7(4):24. doi: 10.3390/children7040024 (PMC7231028; doi:10.3390/children7040024)
Supplement: Supplementary file 1 [file children-07-00024-s001.zip › Supplementary table S1.docx]

**Supplementary table S1:** Dietary intake frequency questions

| Survey question | Frequency | Children | Parents |
| --- | --- | --- | --- |
| How many serves of fruit? [mean ± SD] |  | 0.65 ± 1.1 | 0.5 ± 0.5 |
| How many serves of vegetables? [mean ± SD] |  | 0.94 ± 1.6 | 1.76 ± 1.77 |
|  |  |  |  |
| How often do you eat fruit? | Always:  Most of the time:  About half the time:  Sometimes: Never: | 10 (47.6%) 8 (38.1%) 3 (14.3%) - - | 13 (61.9%) 3 (14.3%) 1 (4.8%) 4 (19%) - |
| How often do you eat vegetables? | Always:  Most of the time:  About half the time:  Sometimes: Never: | 8 (38.1%) 8 (38.1%)  3 (14.3%)  2 (9.5%) - | 15 (71.4%) 6 (28.6%) - - - |
| How often do you drink water? | Always:  Most of the time:  About half the time:  Sometimes: Never: | 19 (90.5%)  1 (4.8%) 1 (4.8%)  -  - | 19 (90.5%) 2 (9.5%) - - - |
| How often do you consume milk and milk products? | Always:  Most of the time:  About half the time:  Sometimes: Never: | 7 (33.3%)  8 (38.1%)  4 (19.0%) 2 (9.5%) - | 10 (47.6%) 5 (23.8%) 2 (9.5%) 4 (19.0%) - |
| How often do you consume meat/tofu and fish? | Always:  Most of the time:  About half the time:  Sometimes: Never: | 5 (23.8%)  9 (42.9%)  3 (14.3%)  4 (19.0%) - | 8 (38.1%) 5 923.8%) 7 (33.3%)  6 (28.6%) - |
| How often do you consume bread? | Always:  Most of the time:  About half the time:  Sometimes:  Never: | 7 (33.3%)  10 (47.6%) 2 (9.5%)  2 (9.5%) - | 7 (33.3%) 4 (19.0%) 3 (14.3%) 6 (28.6%) 1 (4.8%) |
| How often do you consume potatoes, pasta, rice? | Always:  Most of the time:  About half the time:  Sometimes: Never: | 4 (19%)  8 (38.1%)  4 (19%)  5 (23.8%) - | 3 (14.3%) 5 (23.8%) 7 (33.3%) 5 (23.8%) 1 (4.8%) |
| How often do you consume biscuits? | Always:  Most of the time:  About half the time:  Sometimes: Never: | -  1 (4.8%)  2 (9.5%)  14 (66.7%) 4 (19%) | - 1 (4.8%) 2 (9.5%) 15 (71.4%) 3 (14.3%) |
| How often do you consume lollies? | Always:  Most of the time:  About half the time:  Sometimes: Never: | -  -  1 (4.8%)  17 (81.0%) 3 (14.3%) | - - - 12 (57.1%) 9 (42.9%) |
| How often do you drink soda? | Always:  Most of the time:  About half the time:  Sometimes: Never: | -  -  -  6 (76.2%) 5 (23.8%) | - 1 (4.8%) - 4 (23.8%) 15 (71.4%) |
| How often do you drink ‘diet’ soda? | Always:  Most of the time:  About half the time:  Sometimes: Never: | -  -  -  3 (14.3%) 17 (81.0%) | 1 (4.8%) 1 (4.8%) 5 (23.8%) 14 (66.7%) |
|  |  |  |  |
| How often do you have meals or snacks such as burgers, pizza, chicken, or chips from places like McDonalds, Hungry Jacks, Pizza Hut, KFC, Red Rooster or local takeaway food places? | Never:  Less than once per month:  1-3x per month:  1x per week: 2x per week: 5x per week:  I don’t know: | 1 (4.8%)  10 (47.6%)  5 (23.8%)  4 (19%)  - - 1 (4.8%) | 5 (23.8%)  5 (23.8%)  8 (31.8%)  3 (14.3%)  - - - |
| How often do you eat hot chips, French fries, wedges, fried potatoes or a packet of chips? | Never:  Less than once per month:  1-3x per month:  1x per week: 2x per week: 5x per week:  I don’t know: | 2 (9.5%)  2 (9.5%)  9 (42.9%)  5 (23.8%)  1 (4.8%)  1 (4.8%)  1 (4.8%) | 2 (9.5%)  5 (23.8%)  12 (57.1%)  2 (9.5%)  - - - |
| How often do you eat lollies without chocolate? (for example: lollipops, snakes, skittles, starbursts) | Never:  Less than once per month:  1-3x per month:  1x per week: 2x per week: 5x per week:  I don’t know: | 2 (9.5%)  2 (9.5%)  10 (47.6%)  3 (14.3%)  2 (9.5%)  1 (4.8%) | 8 (38.1%)  9 (42.9)  3 (14.3%)  1 (4.8%) -  - - |
